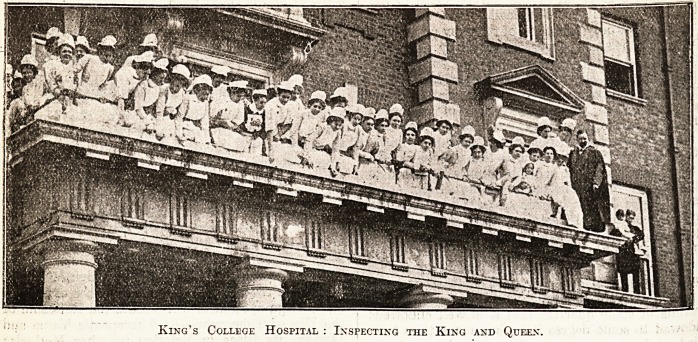# Hospital and Institutional News

**Published:** 1913-08-02

**Authors:** 


### HOSPITAL AND INSTITUTIONAL NEWS.

### POLITICIANS AND THE HOSPITALS.

The full report of the discussion of the Parliamentary Committee on Insurance on Tuesday, which we publish elsewhere, gives point to the warning by Sir Henry Burdett at the Brighton meeting of the British Medical Association. At the Oxford Conference of the British Hospitals Association several provincial hospital officers claimed that under Clause 12 the voluntary hospitals might benefit, by an arrangement with approved societies, from the admission of insured persons having no dependants. It will be seen that this view must be changed in consequence of Mr. Masterman's amendment which, the *Times* states, was forced upon the Committee without notice. This amendment will form a new clause in the Amending Act, and if it is retained we fear the possibility of the hospitals receiving any payment from insured persons under the Act will be gravely diminished, if not entirely destroyed. The evident aim of Mr. Masterman's amendment is to provide a substantial sum in cash for the in-patient, being an insured person, after leaving a voluntary hospital—that is, a dole in fact—without provision for a portion of it to be handed over to the hospital authorities towards the cost of the highest and most costly form of special treatment which the patient may receive. Mr. Lawson properly described Mr. Masterman's new clause as a backward step whereby Parliament was going to decrease "the resources of the great London hospitals especially, which really advance the science of therapeutics upon which the health of the country depends." This will be a great dis-service to the working classes and the new clause enforces Sir Henry Burdett's warning that party politicians are becoming the curse of the sick. Nothing more amazing has probably fallen from the lips of a politician than the declaration of Mr. Masterman that this new clause, instead of doing any injury to the hospitals, in so far as it will have any effect at all, will benefit them. He thus shows gross ignorance or complete indifference to the claims of the voluntary hospitals. Yet without their generous aid National Insurance in this country under the present Act would be financially dead. We look to the members of the House of Commons apart from all party considerations to put Mr. Masterman right and to secure justice to the hospitals and the sick in the best interests of the artisan class.

### THEIR MAJESTIES VISIT NEW "KING'S."

With a conspicuous absence of the pomp and circumstance of high occasions of State, the King and Queen last Saturday paid a visit to the new King's College Hospital which has been steadily rising from the ground at Denmark Hill. In an open carriage without escort—just one single constable riding ahead—they drove through Kennington and Camberwell to the hospital heartily cheered all the way by crowds consisting largely of those who by personal experience can appreciate the blessings of the modern voluntary hospital. In the presence of a large assembly of guests the King declared the hospital open, adding a sentence of good wishes for the work and welfare of the hospital. It is significant of the complex organisation and construction of a large hospital nowadays that their Majesties spent over an hour inspecting the hospital, and even then saw only parts of it. Yet the buildings finished and ready for inspection are only a portion of those which must be made available for patients before the doors are opened to the sick in October; and, in turn again, the buildings then put in commission will provide for but 224 cases, as against the 600 whom the completed design will accommodate. It will thus be realised that colossal labours still confront the managers before their fine scheme can be anywhere near complete, and it will, we are afraid, be some years before this consummation can be expected.

### THE PRESENTATION OF PURSES.

The opening ceremony took place in the out-patients' waiting hall, a lofty room capable of seating five hundred patients. Here also about a hundred children wearing wild roses in turn presented purses to Her Majesty the Queen for the hospital building fund. In all the quite "useful" total of £3,700 was thus received. Several unrehearsed incidents in this part of the ceremony created considerable amusement; high dignitaries of the Church were seen saving infantile courtiers from nasty tumbles and some of the mites were quite loth to part with the purses which they had to present to the Queen. During the subsequent tour of inspection their Majesties were shown observation wards, consisting of separate glass-screened cubicles whereby the nurse in charge can watch the patient with the least possible exposure to contagion; rooms for maniacal patients, with thickly padded walls; engine and boiler rooms; kitchens; and some of the main wards. After that they visited the nurses' quarters, where the architect has provided a separate bedroom for each nurse in accordance with the best modern practice, and finally took tea in the board room, where hangs the portrait of the great Lister himself. Thus ended a memorable afternoon which just missed being the last as well as the first of the hospital's existence. For the next day an alarm of fire was raised and twelve fire-engines were quickly on the scene; fortunately the fire was not in the main block, but in a builder's shed, and it was soon got under control.

### KING'S COLLEGE HOSPITAL AND ITS ARCHITECT.

The new King's College Hospital buildings reflect great credit upon the architect, Mr. W. A. Pite. The architect's work has been extremely well done, and wherever he has had a free hand, which is not generally the case, as the whole scheme of the buildings was practically settled before the competition, the architect's marked ability and readiness to assimilate new ideas and to weigh pros and cons with knowledge is frequently apparent. It was a great refreshment to find evidence of these traits in a new hospital of the size and importance of that at Denmark Hill, seeing that architects have too frequently reproduced the pavilion type without exhibiting the knowledge and conscientiousness to bring each unit fully up to the modern standard, especially in important matters of detail. It will not, of course, be possible to judge this hospital fully on its merits and to indicate accurately its exact position in relation to modern construction until the buildings are completed and occupied. The concentration in the administration building, the grouping of the whole of the female staff, though necessarily separated into departments, in one building, of which the lady superintendent's offices constitute the centre, is a distinctly good feature of the plans, making for centralisation which we have little doubt will facilitate the work. The wards are well proportioned and structurally have several good points. The windows are especially noticeable in this connection. Many points in the arrangements are novel, and we welcome the fact that Slater's fittings have been so largely selected for the kitchen and other departments.

### A NOTABLE ELEVATION FOR THE NEW HOSPITAL.

The elevation, though simple and relatively inexpensive, is striking and attractive. Mr. Pite has shown notable ability in massing his stone-work and the effect is distinctly good. We welcome this addition to the attractiveness of the façade and exterior, especially of these new hospital buildings. They show what ability can accomplish, and lend force to the argument that beauty is attainable without extravagance in this type of building, providing the necessary brains are available. A good deal of ingenuity is exhibited in securing light for the communication corridor in the out-patient department especially. The general effect of the entrance hall, corridors, and many portions of the interior of the buildings adds new attractiveness to this, our latest palace of healing. Every visitor with a knowledge of the history of hospitals and their evolution will probably be somewhat startled on entering the chapel, which is so unusually spacious as more to resemble a church of the type frequently met with in foreign hospitals of a much earlier type. We shall be interested to know how far, and to what extent, this large building is fully availed of by the patients and the staff. Apart from the architect, one or two matters may be noted. First of all the site, twelve acres, is too small, having regard to the many buildings which are to be erected upon it to complete the whole scheme. This aspect of the buildings can be more readily appreciated at the present time than probably later, when the hospital is in full work. The architect has shown marked ability, however, in making the best of the site as it stands. We fear that less than complete wisdom has been shown by those responsible for the details of the plans for the out-patient and casualty departments, especially in regard to the operation theatre and the ophthalmic department. These faults will make themselves felt when they are brought into active use. Indeed, we expect that those who habitually work in the ophthalmic department will have grave cause for serious complaint.

### THE KING AND A HOSPITAL BENEFIT PERFORMANCE.

The Chairman and Managing Director of the London Coliseum, Mr. Oswald Stoll, has been informed, it is announced, through the Hon. Sir William Carington, that the King and Queen have granted their patronage to an entertainment which will take place at the London Coliseum on the evening of October 11. The performance is in aid of the French Hospital in London and of the Charing Cross Hospital, and their Majesties hope to be present. The arrangements are being undertaken by Madame Sarah Bernhardt, with whom, it is stated, the idea originated. Madame Bernhardt's tireless energy in charitable endeavour on the stage is almost as remarkable as her unique and time-defying personality itself. She has organised this performance to express in a practical manner her thanks to the people of our nation for the tribute presented to her last year, and is preparing a message to the public in which she will outline the details of her scheme for helping these two hospitals. In her hands and those of Mr. Stoll the programme cannot fail to be of the choicest; and the presence of their Majesties will complete the *éclat* of the occasion, and, we trust, ensure a striking financial success. We congratulate both institutions on the prospect of a bumper benefit night, and on the proof of their Majesties' interest in the work which they are carrying on. Applications for seats should be addressed to the Countess of Lonsdale at Charing Cross Hospital.

### THE EAST LONDON HOSPITAL FOR CHILDREN.

On a previous occasion we drew attention to the difficulties which the governors are encountering in their efforts to make ends meet at this institution, familiarly known as "Shadwell." The half-yearly report, presented last week to the Court of Governors, shows that things are getting desperate. Last year the hospital kept going by means of one exceptionally large donation and of legacies; but this year there has been an enormous decrease in receipts, with a heavy rise in expenditure. The total of the latter for the last six months is £6,458, whereas the former amount only to £2,707. Such a ruinous ratio cannot long go on, of course, and the board are discussing the sale of investments, which at the present time would involve a heavy sacrifice. We are well aware of the valuable work which this institution carries on in East London, and the difficulty of raising money for it, owing to the lack of any affluent people within some miles of it. Notwithstanding the loss it would be to the poor of Shadwell, we feel it would be sounder policy to cut down expenses by closing some of the wards than to sell out investments, especially in the depressed state of the market. The figures of income and expenses given above are surely sufficient evidence that financial disaster is inevitable unless either income can be increased or expenses lessened; and the latter object can be attained by a stroke of the pen, if our suggestion is adopted.

### WINDFALLS FOR HOSPITALS.

On July 25 the new buildings of the Hospital for Epilepsis and Paralysis, Maida Vale, were opened by Princess Louise, Duchess of Argyll. On the previous day a gentleman walked into the hospital, and, refusing to give his name, handed a bank note for £1,000 to the Secretary, Mr. Burleigh, to be placed to the building fund account. This generous gift left only £2,500 more to be collected in order to liquidate the debt on the buildings; and we trust this anonymous donor's liberality may stimulate other friends of the hospital to help in raising this remaining sum. By the will of the late Mr. J. N. Mappin the following hospitals will receive legacies of £1,000 each: The London Hospital, Royal Free Hospital, The Queen's Hospital for Children, Chelsea Hospital for Women, St. John's Hospital for Diseases of the Skin, Royal Hospital for Incurables, Epsom Cottage Hospital, Leatherhead Cottage Hospital, Cobham Cottage Hospital, Royal Sea Bathing Hospital (Margate), Royal Hospital, Sheffield, and Sheffield Infirmary.

### THE DOWNS SANATORIUM COMPLAINTS.

It is announced that a Sub-Committee of the London Insurance Committee has been directed to investigate the above during the recess, and, indeed, such a course was imperative in view of the recent suicide of a patient. With the facts *sub judice* it is clearly not profitable to make much comment just now, but from what transpired at the meeting when the above decision was arrived at it might first be said that shortcomings in the commissariat department do not seem a very likely cause. The Metropolitan Asylums Board, the body administering the Downs Sanatorium, has a very long and large experience in this direction, and a particularly complete, even meticulous, organisation of food supplies. What will strike the experienced tuberculosis worker is that a wrong tone seems to have been taken by the immediate management towards the patients. A man found smoking in bed "refused to express regret." He should not have been given the chance to do so. A minor punishment should have been immediately awarded, with the warning that a second offence would lead to his dismissal at twenty-four hours' notice. The others who broke bounds and invaded a public-house should, in place of being asked to "abide by the rules," have been packed off. How can it have been possible for a patient the morning after he arrived to call a mass meeting of his companions, or for another to refuse to go when dismissed, if proper discipline of patients had existed? It was observed in these columns that when the Metropolitan Asylums Board appointed one of their own employees, and not an experienced tuberculosis administrator, to the charge of their first sanatorium that they were taking a rather large risk, and although we have no wish to prejudge the case, or to imply that sudden and severe trials may not spring up in any institutional management, yet we think it right to make the above comments. In this connection some useful reflections may be found in an article to be published in The Hospital entitled "Beginners' Mistakes in Tuberculosis Work." It is a commonplace that the most successful sanatorium residents have been martinets rather than otherwise, and under their rule there was no chance of a general loss of moral tone leading to rebellion on the part of more active spirits and despondency in the weaker ones.

### INSTALLATION OF X-RAYS AT ALNWICK INFIRMARY.

The opening of the *x*-rays department of the Alnwick Infirmary took place on July 22 in the presence of a numerous gathering. The apparatus is the last gift of the late Duchess of Northumberland to the Infirmary. Major Widdrington, of Newton Hall, senior trustee, occupied the chair. In the course of his remarks he said the late Duchess had left a legacy of inestimable value in the record of a well-spent life. In the midst of her busy and active life in other spheres she never neglected what he believed to have been the chief characteristic of her life, the wish to alleviate suffering—suffering in whatever rank or circumstances of life she found it, whether in mind, body, or estate. The Chairman ordered a plate to be affixed to the chamber bearing the following inscription: "This Röntgen rays apparatus was presented to the Alnwick Infirmary by Edith, Duchess of Northumberland, on June 29, 1913, for the prevention and relief of suffering in Alnwick and the surrounding district." A demonstration of the *x*-rays was given by Dr. Scott Purves and Dr. R. B. Robson.

### WORCESTER GENERAL INFIRMARY.

At the monthly meeting of the executive committee the report of the sub-committee (appointed to consider the details necessary for carrying out the recommendations with regard to the financial crisis) was presented. The report suggested the retrenchment of one resident medical officer, two sisters, six nurses, three maid-servants, and one porter. The honorary medical staff recommends that the salaries of the chaplain, matron, and the dispenser be reduced by 10 per cent., and that of the secretary by 20 per cent. It was estimated that the approximate annual saving thus effected would be about £2,000. Alderman Leicester, in expressing his deep regret at the state of things they had to face, commented on the absence in the report of any suggestion for increasing the income, either by increasing the price of letters, or by charging the patients who could afford to pay. It was stated, in reply, that the sub-committee was not instructed to deal with that question; also, that when all these economies should have been effected the income would still be £1,000 or £2,000 below the required amount. There was more discussion, and it was agreed to re-appoint the sub-committee after the meeting of the governors, with a view to devising means whereby the income of the institution can be increased.

### HOSPITAL EXTENSIONS IN THE MIDLANDS.

On the same day, July 17, two important extensions were on view in the Midlands. The North Staffordshire Infirmary has undertaken alterations which have cost in all £35,000; the children's ward, completed at a cost of £3,000, was formally opened on that day by Lady Stamer, and marks the conclusion of this ambitious scheme. The new ward contains thirty-two cots, and has been rendered possible only through the generosity of Mrs. Spibey, who, however, declined to perform the opening ceremony. It was stated that the institution is in need of a £3,000 annual income for upkeep. The other extension is the new King Edward VII. Memorial Wing of the Coventry and Warwickshire Hospital, now practically finished, and thrown open for inspection on July 17. This is part of a £15,000 scheme, designed ultimately to enlarge the hospital to a capacity of 145 beds. The Mayor and Mayoress and other leading citizens of Coventry were invited by the Chairman of the hospital, Mr. Iliffe, and after inspecting the building were entertained at tea on the hospital balcony; a practical demonstration of open-air treatment in a hospital was thus afforded to the guests.

### A QUESTION OF APPORTIONMENT AT SHEFFIELD.

An interesting discussion, initiated by Dr. Pye-Smith, has taken place at Sheffield concerning the basis of apportionment among the voluntary hospitals of that city of the funds collected by means of the local Hospital Sunday Fund. This year the sum for division amounted to £2,136; of this the Royal Infirmary took £1,026, the Royal Hospital £654, the Jessop Hospital £336, and the Children's Hospital £118. This distribution was based solely on the relative expenditures of the four institutions, and Dr. Pye-Smith's argument was that this is not the best system because it holds out no inducements to economy. He suggested that in future some more rational method of apportionment should be adopted, based on a consideration of the number of patients or the number of occupied beds. Another speaker, Mr. Wake, agreed that the present system is not a good one, but pointed out the difficulties of the subject. The Jessop Hospital, for instance, has nothing like the out-patient expenses of the two larger hospitals; so that a distribution based solely on in-patients would favour the Jessop unduly. He brought forward a suggestion that the chairmen of the four hospitals should meet together to discuss the matter. Eventually Dr. Pye-Smith expressed a desire not to move further in the matter; but the question he has raised is one of much importance and can hardly be dropped altogether. We think that the committee of the Sheffield Fund would do well to approach the Metropolitan Hospital Sunday Fund authorities and take a lesson from them in the problems of distribution and apportionment.

### THE WESTMINSTER—ST. GEORGE'S AMALGAMATION.

A quarterly and special general board of governors of Westminster Hospital was held on July 23 to consider the proposed amalgamation with St. George's, details of and comments upon which have been published in The Hospital, July 19, 1913, pp. 461 and 483. It was, we may reasonably infer, a foregone conclusion that proposals so advantageous would be carried without difficulty; for the decision to remove from Broad Sanctuary had already been taken months ago, so the sentimental objection had been overcome. As it turned out, the resolutions were carried unanimously. Essentially they were upon parallel lines with the resolutions passed a fortnight before at St. George's. The first of the four resolutions. "that an amalgamation of Westminster Hospital with St. George's Hospital is desirable," contained the whole crux of the matter; and the other three resolutions were concerned with the ways and means of the operation thus indicated. To all intents and purposes the first steps which count have now been taken, and the fruition of this fine conception is now only a matter of time.

### ANOTHER ASPECT OF HOSPITAL REMOVAL.

Ever since the decision to remove St. George's Hospital from its present site was made known, primarily in these columns, discussion has been active concerning the results to the inhabitants of Battersea and Belgravia respectively, to the students and the staff. Another aspect of the proposal has attracted less attention, and that is the effect upon neighbouring hospitals. Some of the accident cases in Piccadilly will no doubt be taken to Charing Cross Hospital, in Kensington to the West London, and in the park area to St. Mary's. The Editor of the *St. Mary's Hospital Gazette* looks a little further still into the future, and perceives quite clearly that many emergencies of all kinds will seek aid at St. Mary's which would be treated at Hyde Park Corner if St. George's were still there. He states that even now many simple fractures of the leg have perforce to be passed on to the infirmaries, and that the waiting list for non-urgent surgical cases is terribly long. So that any marked increase in the number of accident cases and acute abdominal emergencies admitted to St. Mary's will prevent the admission of a corresponding number of non-urgent cases. When, if ever, St. Mary's adopts on a large scale the teachings of Sir Arbuthnot Lane as to the operative treatment of simple fractures, this tendency will be even more marked. It will thus be seen how complicated are the issues raised by this removal project, necessary and advisable though we believe it to be.

### ST. LUKE'S HOSPITAL.

Naturally enough, the name of the "good physician" has been frequently adopted whereby to designate institutions for the relief of sickness and suffering. One of the oldest of these foundations, as well as one of the best known and most useful, is St. Luke's Hospital in Old Street, close to the City Road, for mental disorders. Yet we would guarantee that only a fraction of the busy population which daily passes the somewhat gloomy old building knows what it is or the work it does. St. Luke's is one of the few hospitals which really do help that most deserving of all classes, those who are too poor to pay the expenses of prolonged illness or of major surgery, yet not poor enough to be fit subjects for public rate-supported institutions or free hospitals. For the middle classes it supplies a want, or helps to supply it, which is most keenly felt. The responsibility of a case of insanity in a family of this class is too great for any but institutional treatment; and yet the feeling against a pauper asylum is very real and very proper. St. Luke's does a work the importance of which is not recognised as it should be; and its position puts it somewhat out of court in attempts to catch the public eye. Again, an impression prevails in some quarters that it is well endowed; endowed to some degree it is, but more than two-thirds of the expenses have to be met by subscriptions and donations. We are very glad to see that the needs of this fine old hospital have been placed before the public in the columns of the *Evening News*, and to express our appreciation of the extremely fair and readable article which a well-known journalist wrote for that paper. May it bring in the much-needed support!

### THE MOUNT VERNON HOSPITAL STAFF.

It is not with any desire to stir up the already sufficiently grave controversies between the governors and the medical staff of the Mount Vernon Hospital that we allude again to the staff changes at this institution. A fortnight ago (The Hospital, July 19, 1913, p. 462) we noted the resignation of the majority of the medical staff, and dealt with the dispute which underlay it. We now note that the governing body are advertising for a surgeon, a surgeon laryngologist, and an anæsthetist. Curiously, no mention is made of any vacancy for physicians or assistant physicians: possibly those who did not resign are considered sufficient to carry on the work of the institution until the expansion contemplated at the Northwood branch is brought about. One rather striking item in the announcements of these vacancies deserves mention. For the post of anæsthetist, candidates must, it is stated, be registered medical practitioners; and, it is added, Fellows of the Royal College of Surgeons will be preferred. The London anæsthetists who possess this qualification could certainly be counted on the fingers of one hand: from memory we can think of only two. Nor can it be argued with any force that the training required for this diploma especially fits a man to be an anæsthetist. We do not recollect ever seeing or hearing of such a clause in relation to a post as anæsthetist before; and we think it a foolish and unnecessary one. It will so narrow the field of selection as to deprive the governors of any but the most restricted choice. If that is the object in view, there was no need to advertise the appointment at all; it could have been made just as well privately (unless the laws of the hospital compel the public advertisement of vacancies). If that is not the object, we fail to see any sense in the clause at all.

### THE PREVENTION OF CONSUMPTION.

The fifth annual Conference of the National Society for the Prevention of Consumption is to be held at the Central Hall, Westminster, on August 4 and 5, when a full programme will be got through. On the first day the Prime Minister is to deliver the Opening Address. After that, Tuberculin Treatment becomes the subject of the day's work: a general survey of this topic by Dr. H. W. G. Mackenzie is to be followed by Professor Sims Woodhead and Professor Rabinowitch-Kempner on the Nature and Preparation of Tuberculin. In the afternoon a large number of British and foreign specialists will discuss the Rationale of the Use of Tuberculin—its Therapeutic Value and Dosage; its Place in Relation to other Methods; and its Range of Application. On the second day of the Conference there is to be a public discussion on the Need for the Co-ordination of Anti-Tuberculous Measures. This will be opened by Sir R. W. Philip, and representatives of most of the leading countries of Europe will take part in it. At four o'clock Dr. Inman will give a demonstration of the Preparation and Properties of Tuberculin. In the evening of the first day, August 4, the annual meeting of the Association will take place, followed by a conversazione at which cinematograph illustrations of the fight against consumption will be shown.

### INSANITARY RAILWAY CARRIAGES.

The St. Pancras Borough Council have adopted a recommendation of the public health committee, that the local members of Parliament should be urged to consider favourably a suggestion that action should be taken by the sanitary authorities in London with regard to the insanitary arrangements on the majority of English railways. It will be interesting to see what is the next step taken to secure an improvement in these arrangements, which are admittedly very bad and call for drastic remedy. The St. Pancras Borough Council are to be congratulated on raising the question.

### LAMBETH'S CENTRAL DISPENSARY.

Owing to some subsidiary arrangements which have yet to be made, the dispensing work for the four districts of Lambeth will not at the outset be undertaken at the new dispensary erected by the Lambeth Board of Guardians, the work for one district being left to be included at a later date. The building is nearing completion and the appointment of a temporary assistant dispenser has been authorised. Mr. R. Allison, the dispenser at Stockwell, has retired, and the dispenser from Montford House Dispensary has been transferred to Stockwell.

King's College Hospital: Inspecting the King and Queen.

### THE NEW MATRON AT MIDDLESEX HOSPITAL.

Miss M. G. Montgomery, who has held the post of matron at Addenbrooke's Hospital, Cambridge, for the past five years, has been appointed matron of the Middlesex Hospital, in succession to Miss Lloyd Still. Miss Montgomery was trained at St. Thomas's Hospital, and when Sister Chantry there was elected, in May 1908, from a large number of candidates to the matronship at Addenbrooke's. During the five years Miss Montgomery has been at Cambridge she has given ungrudgingly of her time and talents in everything and in every way which made for the welfare of the patients, the nurses, and the institution in general. "And her departure," a correspondent writes, "will be greatly deplored by everyone at Addenbrooke's and in Cambridge. Miss Montgomery possesses all those sterling qualities so essential in her profession and the making of a good matron. She is the very essence of kindness, yet firm—in fact, it has always struck me that she is verily gifted with the good qualities of that great and good Christian lady, Florence Nightingale."

### ALEXANDRA DAY.

The report of the meeting of the administrative committee of the Alexandra Day collections at the Mansion House, published this week, seems to be satisfactory so far as it goes. Unfortunately the figures are not stated in sufficient detail to make them readily understandable. In brief, it would appear that the sum credited to London this year exceeds £24,608, of which we gather between £5,000 and £6,000 represents "sales to provincial cities and towns and percentages on the gross takings in the latter," and £18,378 the cash collections in London. The cash collections in the provinces are stated to amount to £23,069. The expenditure on stock, salaries, postages, out-of-pocket expenses, etc., amount to some £7,500, and the actual money available for distribution in London would seem to be some £14,000. Of this upwards of £6,000 has been given in relatively small sums to a large number of miscellaneous charities, in addition to certain hospitals, and a further £8,000 is to be distributed amongst various hospitals and dispensaries in London. We understand the accounts have not yet been signed, nor can we obtain a copy of them. So far as the figures published can be analysed it would seem as if the expenses on the whole had been kept well in hand providing the figures stated include all the expenditure by the London office for the organisation of Alexandra Day 1913.

### PURCHASE OF RADIUM.

The great advantage of installing the $x$-ray apparatus in an infirmary has been experienced at St. John's Hill Infirmary, Wandsworth. The general desire on the part of Boards of Guardians has been to run their infirmaries as cheaply as possible. At Wandsworth, however, the Guardians have realised that it is a much cheaper and wiser policy to have an up-to-date institution, and the introduction of the $x$-ray has mitigated much suffering and labour. They have decided to ask the permission of the Local Government Board to expend £250 on the purchase of radium, the first request of this kind the Local Government Board has had before it. Should Wandsworth be successful, it will be a precedent and incentive to other Boards of Guardians, who require a striking example of this nature in order that they may be brought to realise their full responsibilities to the sick poor committed to their charge.

### TO OUR READERS.

Contributions are specially invited from any of our readers to these columns. They should deal with topical subjects and news. They must be authenticated for the information of the Editor only. The minimum payment if published is 5s. There is no hard-and-fast rule as to space, but notes of about twenty lines in length are preferred.

## Figures and Tables

**Figure f1:**